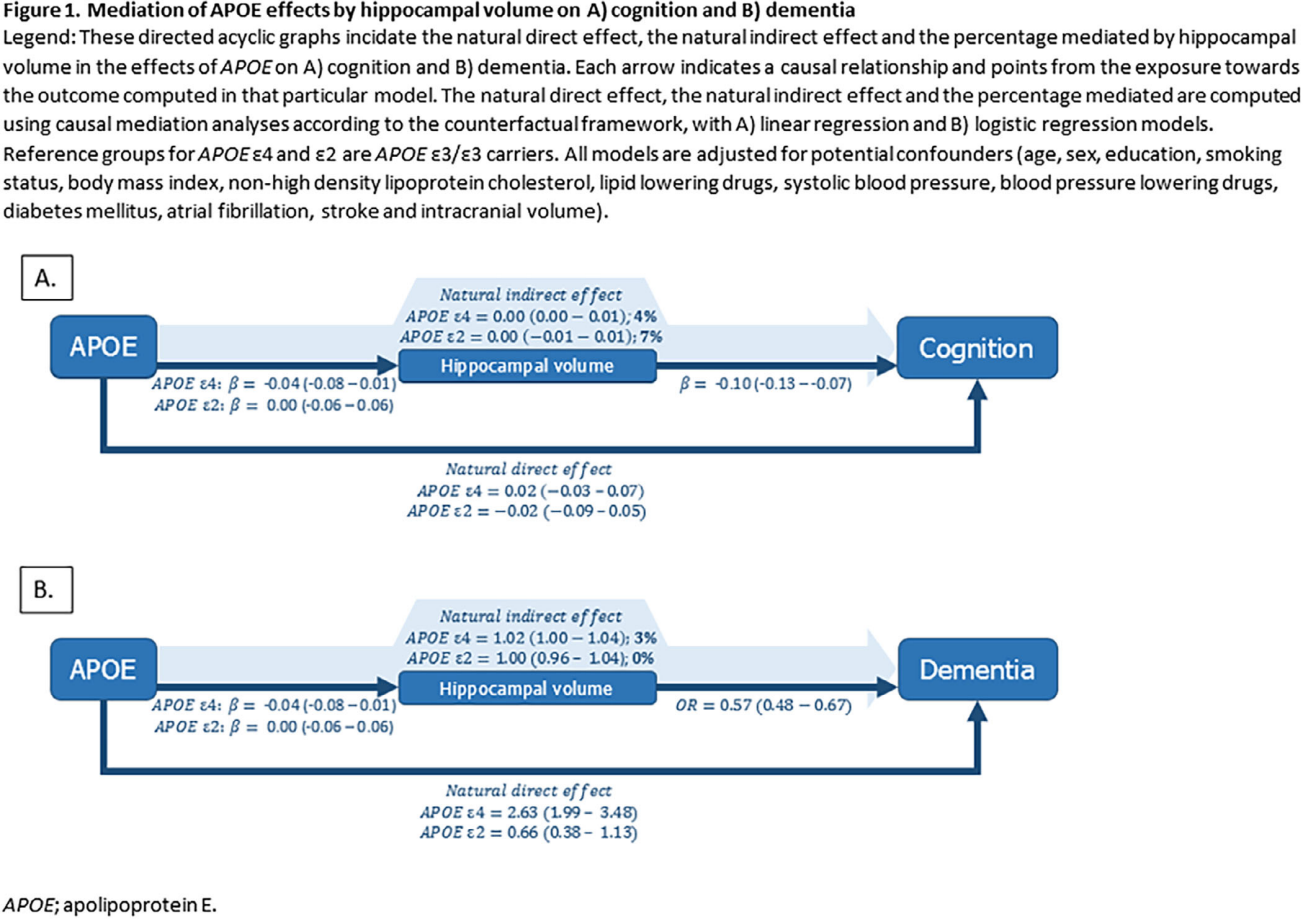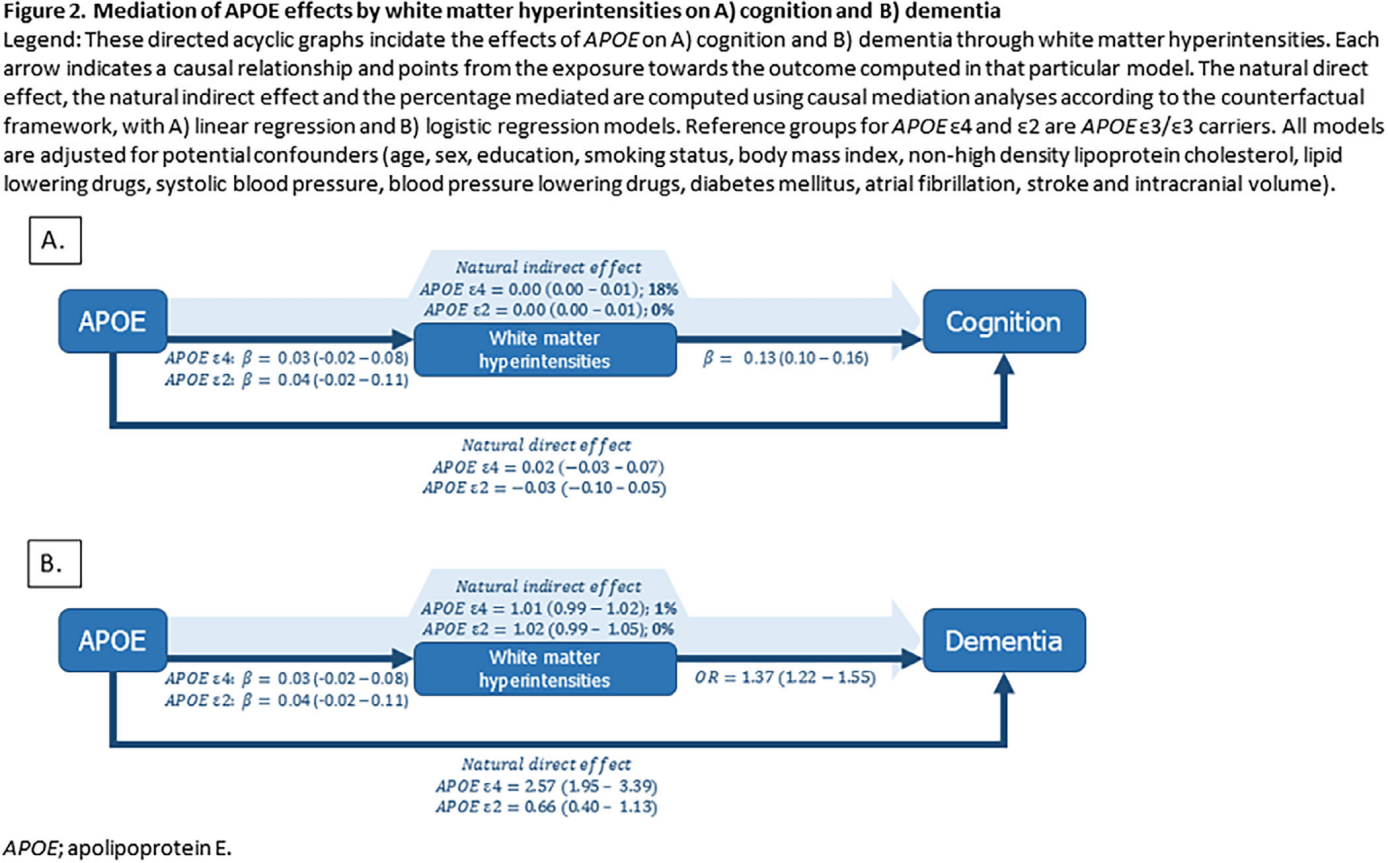# APOE genotype, cognition and dementia: mediation of ε2 and ε4 effects by structural brain imaging markers in a population‐based study

**DOI:** 10.1002/alz.087364

**Published:** 2025-01-09

**Authors:** Jacqueline Josephine Claus, Mathijs T. Rosbergen, Meike W. Vernooij, Frank J. Wolters, Arfan Ikram

**Affiliations:** ^1^ Erasmus Medical Center, Rotterdam, Zuid‐Holland Netherlands; ^2^ Erasmus MC University Medical Center, Rotterdam, Zuid Holland Netherlands; ^3^ Erasmus University Medical Center, Rotterdam Netherlands; ^4^ Erasmus University Medical Center, Rotterdam, Zuid‐Holland Netherlands; ^5^ Erasmus University Medical Center, Rotterdam, Zuid Holland Netherlands

## Abstract

**Background:**

Insight into APOE‐mediated pathways is important to unravel pathophysiology and identify therapeutic targets against late‐life cognitive decline and dementia. However, ante‐mortem studies on the mediated effect of APOE on cognition and dementia through different disease markers on structural brain imaging remain scarce, in particular for APOE‐ε2.

**Method:**

We included all dementia‐free participants from the population‐based Rotterdam Study, who routinely underwent cognitive assessment and brain MRI between 2005‐2009, and were followed‐up for incident dementia until 1‐1‐2020. We standardized hippocampal volume (HV) and volume of white matter hyperintensities (WMH), and performed causal mediation analyses to quantify the mediation effects (i.e., natural direct effect, natural indirect effect and percentage mediated) of APOE ε4 and ε2 carriership on cognition operationalized as g‐factor and incident dementia, with ε3/ε3‐carriers as a reference group. We adjusted all models for potential mediator‐outcome confounders (i.e., demographics, education, cardiovascular factors) and for intracranial volume.

**Result:**

Among 5,510 participants (mean age: 65.0[±10.9] years, 55.0% women), 3,244 were ε3/ε3‐homozygotes, 709 ε2‐carriers, and 1,557 ε4‐carriers. During a median 11 years follow‐up, 349 participants developed dementia, of whom 148/1,557 ε4‐carriers (9.5%), 172/3,244 ε3/ε3‐carriers (5.3%) and 29/709 ε2‐carriers (4.1%). APOE‐genotype was not significantly associated with hippocampal volume (Figure 1), whereas those carrying APOE ε2 or ε4 tended to have a slightly higher volume of white matter hyperintensities (Figure 2). In total, 4% of the effect of ε4, and 7% of the ε2‐effect on cognition were mediated by HV. The percentage of the effect on cognition mediated through WMH was 18% for ε4 and 0% for ε2. For incident dementia, there was little to no evidence of mediation by either HV (ε4: 3%; ε3: 0%) or WMH (ε4: 1%; ε3: 0%), which was consistent in sensitivity analyses stratified by duration of follow‐up and by age (≥75 years).

**Conclusion:**

In this population‐based cohort study, we found that one fifth of the effect of APOE‐ε4 on cognition was mediated by WMH, compared to only 4% by lower HV. For dementia, however, we found no significant mediation effects of both HV and MWH, suggesting the involvement of alternative pathways through which APOE exerts its effect on dementia.